# Enterocytes in Food Hypersensitivity Reactions

**DOI:** 10.3390/ani11092713

**Published:** 2021-09-17

**Authors:** Maja Krstić Ristivojević, Danijela Apostolović, Katarina Smiljanić

**Affiliations:** 1 Center of Excellence for Molecular Food Sciences, Department of Biochemistry, Faculty of Chemistry, University of Belgrade, Studentski Trg 12-16, 11000 Belgrade, Serbia; katarinas@chem.bg.ac.rs; 2 Division of Allergy and Immunology, Department of Medicine Solna, Karolinska Institute and University Hospital, 17164 Stockholm, Sweden; danijela.apostolovic@ki.se

**Keywords:** enterocytes, food hypersensitivity, food allergy, antigens, oral tolerance, CD23, animal models of food allergy

## Abstract

**Simple Summary:**

Hypersensitivity to food, affecting both animals and humans, is increasing. Until a decade ago, it was thought that enterocytes, the most abundant constituent of the intestinal surface mucosa layer, served only to absorb digested food and prevent foreign and non-digested substances from passing below the intestinal layer. Growing evidence supports the involvement of enterocytes in immunological responses. Here, we present a comprehensive review of the new roles of enterocytes in food hypersensitivity conducted in animal models in order to better understand complicated immune pathological conditions. In addition, resources for further work in this area are suggested, along with a literature overview of the specific roles of enterocytes in maintaining oral tolerance. Lastly, it will be beneficial to investigate the various animal models involved in food hypersensitivity to reach the needed momentum necessary for the complete and profound understanding of the mechanisms of the ever-growing number of food allergies in animal and human populations.

**Abstract:**

Food hypersensitivity reactions are adverse reactions to harmless dietary substances, whose causes are hidden within derangements of the complex immune machinery of humans and mammals. Until recently, enterocytes were considered as solely absorptive cells providing a physical barrier for unwanted lumen constituents. This review focuses on the enterocytes, which are the hub for innate and adaptive immune reactions. Furthermore, the ambiguous nature of enterocytes is also reflected in the fact that enterocytes can be considered as antigen-presenting cells since they constitutively express major histocompatibility complex (MHC) class II molecules. Taken together, it becomes clear that enterocytes have an immense role in maintaining oral tolerance to foreign antigens. In general, the immune system and its mechanisms underlying food hypersensitivity are still unknown and the involvement of components belonging to other anatomical systems, such as enterocytes, in these mechanisms make their elucidation even more difficult. The findings from studies with animal models provide us with valuable information about allergic mechanisms in the animal world, while on the other hand, these models are used to extrapolate results to the pathological conditions occurring in humans. There is a constant need for studies that deal with this topic and can overcome the glitches related to ethics in working with animals.

## 1. Introduction

**Scheme 1:**
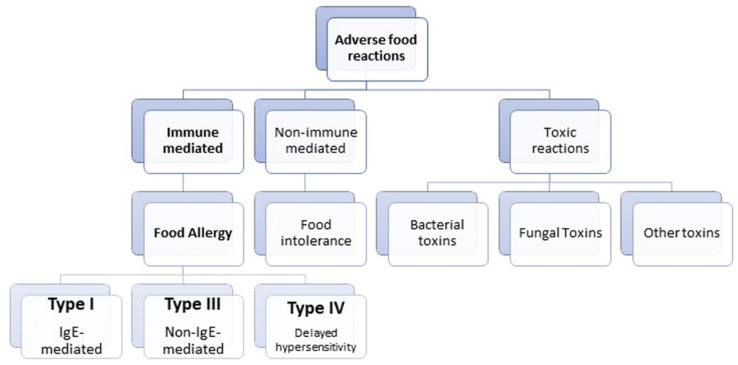
Schematic representation of the classification of adverse food reactions.

Food hypersensitivity reactions belong to adverse food reactions (Scheme 1), which are defined as reactions to an otherwise harmless dietary component. The cause of food hypersensitivity reactions is hidden within derangements of the complex immune machinery of humans and mammals. Food hypersensitivity reactions can be broadly divided into food allergies, food intolerances, and other adverse reactions caused by bacterial toxins (Scheme 1). Food allergies represent aberrant immunological reactions to certain food components, usually a protein. The main focus of this review will be on the role of enterocytes in these allergic reactions or immune-mediated food hypersensitivity reactions (Scheme 1). These abnormal immunological responses could be IgE-dependent, cell-mediated or mixed [1]. In animals with gastrointestinal symptoms, it is often difficult to distinguish between food intolerance and food allergies, while in humans, most adverse food reactions represent food intolerances [2]. Food allergies imply type I, III, and IV immune-mediated hypersensitivity reactions, among which type I is the most studied in human and veterinary medicine [3]. Type I food hypersensitivity reaction is IgE-mediated or immediate hypersensitivity, which occurs when oral tolerance is breached and instead of an IgA immune response in the presence of food antigens, an IgE response is generated. The first phase of this immune response is sensitization when IgE antibodies bind to the peripheral immune cells, mast cells. The second phase is triggered after the second encounter with an antigen and represents the degranulation of mast cells and the release of inflammatory cytokines. Type III hypersensitivity reactions are triggered by the formation of immune complexes and belong to the intermediate hypersensitivity, which starts several hours after the encounter with antigen. Food antigens are not usually considered as type III hypersensitivity provocateurs, but this can be considered as one of the causes of inflammatory bowel disease in dogs [4]. On the other hand, type IV food hypersensitivity reactions belong to delayed hypersensitivity and are described in humans, while there is no precise data about its prevalence in animals [5].

Besides an overview of classifying food hypersensitivity reactions and defining the exact topic, this review paper will cover what is known about the roles of enterocytes in food allergy across different animal species, from both study types, intentionally designed to primarily target animal species as a research goal and those designed to gain knowledge of human food allergy via animal models. Further, the review will address states of oral tolerance and its break, including a description of the underlying “infrastructure” involved, such as the small intestine and enterocytes. Special attention is paid to the role of enterocytes in the modulation of food allergy responses via its low-affinity IgE receptor—CD23 and to enterocytes’ plasticity revealed by the proteomics approach, which is a novel aspect brought into this topic. All of these tend to facilitate understanding of studies done on animal models, whose primary research goal is to reveal phenomena related to enterocytes’ roles in human food allergy. Therefore, this review encompasses a thorough and updated overview of animal food allergy concerning enterocyte’s role(s) in it. Finally, in concluding remarks, new directions and approaches are suggested for more mechanistic research on the roles of enterocytes in animal food allergy.

## 2. Food Allergy across Animal Species

About 8 to 15% of dogs suffer from a food allergy with clinical manifestations more often during puppyhood, which is similar to the human population [6]. The most common food sources of allergens are also shared between dogs and allergic people, such as beef, dairy, wheat, lamb, egg, chicken, soy, oats, and pork. In addition, dogs and humans have similar gastrointestinal tract anatomy and physiology, as well as nutritional requirements [7]. However, even dogs with high levels of IgE are not easy to identify by ELISA and immunoblot experiments, due to intrinsically higher levels of IgG antibody class compared to humans, probably caused by a high parasitic load [8]. In dogs, clinical symptoms of food intolerance and food allergies are almost identical and refer to intestinal allergies, which encompass acute allergic gastritis, food allergies or intolerances affecting the small intestine, eosinophilic enteritis, and allergic colitis [9]. In canine and feline species, food allergies exhibit a wide range of manifestations that are not limited to the skin or the gut only; it could be a combination [10], including respiratory and systemic symptoms [11]. The usual clinical symptoms of gastrointestinal food sensitivity in cats and dogs, such as vomiting, and diarrhea, are often reported [9]. In addition, cutaneous adverse food reactions that are likely to represent food allergies, have a prevalence range of 20–30% among pruritic, allergic, or atopic dogs and half that in cats [12]. However, there is no specific test to confirm the diagnosis of food allergy [13]. Usually, diagnosis is confirmed by dietary elimination/challenges, involving the feeding of previously non-eaten ingredients or dieting with sufficiently hydrolyzed protein sources, followed by oral food challenges, which represent the gold standard in the diagnosis of food allergies manifested via the skin route [9,14,15,16].

In their paper on immediate-type food allergy in humans and domestic animals, Pali-Schöll, et al. [17] comprehensively compared the prevalence of food allergy, its characteristics and symptoms, food sources and allergen molecules, diagnosis, and treatments, by reviewing almost 170 studies [17]. It seems that dogs, cats, horses, pigs, and rabbits are allergic to the food proteins from the Big 8 group (peanut, soy, milk, red meat, fish, nuts, eggs, and wheat), with specificities toward particular molecular species within each food source. Even though there are many more studies in animal food allergy, with a veterinary background (primarily designed to explore animal wellbeing), there is not a single study designed to reveal the characteristics and role of enterocytes in food hypersensitivity reactions in any animal species. The opposite is true for the studies of animal models of food allergy, mostly murine ones.

## 3. Animal Models of Food Allergies

Food allergies are an increasing problem in the human population, so the need for a better and more relevant understanding of the immunological mechanisms of IgE-mediated food allergy has led to the development of different animal models of food allergies. The findings from studies with animal models provide us with valuable information about allergic mechanisms in the animal world, while on the other hand, these models are used to extrapolate results to the pathological conditions occurring in humans. In addition, there is the question of compliance with ethical standards in research work with laboratory animals. It should be emphasized that animals do suffer from food allergies, and more research should be done to highlight the immunological mechanisms of food allergies in animals, which will improve the diagnostics and treatment of food allergies.

According to the general classification, animal models are divided into small and large animal models of food allergy (Table 1). Small animal models include mice, which are generally preferred over large animal models due to their short breeding cycles and manageable housekeeping, as well as relatively easy genetic manipulation compared to larger models [18]. Another small animal model useful for food allergy research is the rat, and different strains are used differently, to study the induction of specific IgE after oral sensitization, to predict the allergenicity of novel proteins, etc. [19]. The large animal models used to study food allergies are dogs, sheep, and pigs. Dogs, like humans, develop allergies to naturally occurring antigens such as house dust mite, pollen [8], and food [8]. Pigs show a similarity in intestinal physiology with humans, and the microflora is more diverse than that seen in rodent models [20]. The advantages of using sheep as animal models of food allergy are their similar size and physiology to humans, as well as calm nature, and fewer ethical restrictions compared to the use of other large animal models [21]. There are three well-characterized canine models of food allergy from which researchers have drawn several conclusions [7,22]. The most important outcome of studies performed on dog food allergy models is the hypothesis that genetically predisposed individuals that suffer from early gut infections are prone to respond to bystander antigens more aggressively compared to healthy individuals. This implies that infections in the intestine lead to an increased passage of the food allergens, which further results in sensitization to otherwise harmless food proteins [23]. The animal models that are used in studies dealing with the involvement of enterocytes in food allergic reactions are summarised in Table 2.

Finally, many highly relevant results have been generated from humanized murine models, but they are not explicitly about further enterocyte involvement in modulating food allergy reactions. A very useful updated overview of the data that humanized murine models have provided us regarding studying food allergy was performed by Kanagaratham, et al. [35], as well as the review work of Huang, et al. [36].

## 4. The Physiological State in an Organism—Oral Tolerance

Oral tolerance can be defined as a default state of unresponsiveness of the immune system toward the luminal constituents, like the innocuous food proteins or commensal microorganisms, thus maintaining intestinal homeostasis. Other routes of exposure to potential inciters of the immune system, via the skin or inhalation, can also induce immune tolerance. The role of the enterocytes in maintaining the concept of oral tolerance is immense since the intestinal barrier is in constant communication and provocation by the luminal food antigens and commensal microorganisms. This provocation refers to ongoing situations in which foreign/luminal substances reaching the intestinal barrier should be recognized as dangerous or innocuous.

A distinction can be made between central and peripheral tolerance. Central tolerance refers to the prevention of a harmful immune response to self-antigens by eliminating developing T and B cells in the thymus and bone marrow. Oral tolerance is a part of the peripheral tolerance to food proteins in the small intestine and relates to local and systemic immune responses. In oral tolerance, two identified effector mechanisms are active suppression mediated by regulatory T cells and clonal anergy or deletion. Which of these two mechanisms will be activated depends on the dosage of antigen; a low dose of antigen promotes active suppression while a high dose of antigen promotes anergy-driven tolerance [48]. An antigen can be acquired directly by intestinal epithelial cells or indirectly, as described in Section 6.2.1 [49]. The important point is that the route of antigen delivery in the small intestine determines the resulting immune responses [50].

The process of establishing oral tolerance in mice starts around the seventh day of postnatal life and ends with the formation of a mature intestinal epithelium with fully functional thigh junctions among enterocytes [51]. The important entities in the initiation and maintenance of oral tolerance are regulatory T cells (Tregs) and intestinal dendritic cells (DCs) [52]. In addition, mucin from goblet cells acts on DCs to render them more tolerogenic [53]. A sub-type of regulatory DCs that express CD103 is responsible for antigen delivery to the draining lymph node and subsequent induction of Tregs [53,54]. These DCs also allow primed Tregs to go back to the lamina propria to interact with macrophages producing IL-10 and expand [53,55]. In addition to Tregs, T cell anergy can also contribute to oral tolerance [56]. The microbiota plays a key role in the development of oral tolerance, through the regulation of macrophages and innate lymphoid cells that contribute to the regulatory phenotype of gastrointestinal DCs [57]. The absence of microbiota is connected to susceptibility to food allergy [53], while the presence of *Clostridia* strains can suppress the development of food allergy through enhancement of Tregs and intestinal barrier function [58]. A study by Zimmer, et al. [59] aimed to examine the mechanism of oral tolerance induction by monitoring the transport of allergen ovalbumin in germ-free mice BALB/c and severe combined immunodeficiency disease (SCID) mice with a genetic immune deficiency that leads to a lack of mature B and T cells. The results showed that ovalbumin is directed to MHC class II-positive late endosomes of enterocytes in BALB/c mice that undergo normal tolerance induction after a single antigen administration. In addition, ovalbumin was found colocalized with MHC class II antigens on the basolateral membrane of enterocytes in BALB/c mice, which is necessary for recognition by CD4^+^ lymphocytes. At the same time, ovalbumin did not cause oral tolerance induction in enterocytes of SCID mice, which were unable to create a transferable tolerogenic moiety after a feed of ovalbumin. These results suggest that the targeting process within the enterocytes is one of the key requirements for the induction of oral tolerance. Additional studies are necessary to examine if targeting of allergens to MHC class II-positive late endosomes within enterocytes underlays induction of oral tolerance [59]. The elucidation of key pathways in oral tolerance, most likely in murine models, could identify new strategies to increase the efficacy of immunotherapy treatments for food allergy [53].

## 5. Break of Oral Tolerance

Failure to generate oral tolerance is the basis of the pathogenesis of the food hypersensitivity reactions. Moreover, the intestinal epithelium is the place of action of the allergic effector cells, which results in the symptoms of food allergy, like diarrhea [60]. There is great interest in discovering the cause of the break of oral tolerance since it would give us the cause of food allergies and possible treatment strategies. Adjuvants such as cholera toxin (CT) is known to break oral tolerance. CT is the soluble toxin that consists of two proteins, the subunit A (CTA), which is present as a monomer in the complex, and the subunit B (CTB), which forms a pentamer [61]. Interestingly coadministration of food antigens and CTB did not induce allergic sensitization [62], which implies that enzymatic activity of CTA is required for the break of oral tolerance, although the target cell is still unknown. The mechanism of the break of oral tolerance by CT and *Escherichia coli* heat-labile toxin (LT) is the subject of many studies and one of them suggests that these adjuvants’ effects are mediated by T cells and antigen-presenting cells (APCs), which results in an IL-4-dependent or -independent immune response [63]. Other ways of breaking oral tolerance are through the interference of the physiological digestion process [64] and physiological stress [65].

Another important problem is how to explain the differences in the potency of different food antigens in the induction of allergic reactions. One of the conclusions is that the protein structure itself is a decisive factor for the magnitude of the allergic response. The structures such as glycans on the protein antigens can be directly recognized by the receptors on DCs (DC-SIGN) leading to T_H_2 skewing through modulation of the DCs phenotype [66]. This means that allergens can behave as self-adjuvants because they can directly bind to pattern recognition receptors on the APCs. Moreover, food processing influences the allergic potential of antigens [67,68,69].

### Enterocytes in Inflammation and upon Break of Oral Tolerance

Again, enterocytes are actively involved in the processes occurring during inflammation and immune response to food antigens upon the break of oral tolerance. In this pathological state, enterocytes actively secrete chemokines which further attract the immune effector cells in the intestinal mucosa [70]. In addition, enterocytes secrete cytokines such as thymic stromal lymphopoietin (TSLP), which is a highly pleiotropic cytokine. This means that TSLP can act as a potent inducer of the pro-allergic T_H_2 response by stimulation of DCs, mast cells, T and B lymphocytes, and can be involved in suppressing inflammatory T_H_1 and T_H_17 responses [71]. Another highly pleiotropic cytokine secreted by the enterocytes is IL-15, which is, under normal conditions, expressed at a low amount on the villus of the enterocytes, while inflammation triggers a high expression of IL-15 in the epithelial layer [72].

## 6. Overview of Morphological Organization of the Intestine

### 6.1. The Small Intestine

The small intestine represents the ductus, which starts from the pylorus of the stomach and ends with the ileocolic valve. The small intestine consists of three anatomical segments, the duodenum (proximal), the jejunum (intermedial), and the ileum (distal). Generally, the length of the small intestine is proportional to the size of the animal. Its length is approximately 1 to 1.5 m in adult cats, and from 1 to 5 m in adult dogs [73]. Microflora, mucosa, and gut-associated lymphoid tissue are the “building blocks” of the small intestine [73].

The intestinal microflora or gut microbiome is an essential part of the small intestine. It consists of many symbiotic microorganisms, which have an inevitable part in the maintenance of mucosal immunity. Studies on germ-free mice showed that their capacity for production of cytokines and the levels of serum immunoglobulins are reduced, and the number of intraepithelial lymphocytes is decreased [74,75,76].

The layer that separates the microflora and intestinal epithelial layer is the mucus layer. It acts as a protective sieve that enables constant exchange between epithelial cells and the lumen of the gut, securing nutrition and prevention of intruder’s breach simultaneously. It is made up of secreted mucus and the glycocalyx, namely the glycoprotein and glycolipid layer covering the cell-surface membranes of enterocytes [77].

The small intestine mucosa presents the largest absorptive surface in humans and animals and consists of the intestinal epithelium and lamina propria, and below which, the submucosa and muscle layer are located. This important feature is illustrated by the fact that the surface of the human intestine is approximately 175 m^2^. It is wisely packed by specific folds of the mucosal wall composed of villus projections toward the intestinal lumen and further perplexed by the presence of the microvilli on the lateral side of the enterocytes, which form the intestinal monolayer. In animals such as cats and dogs, the length of the villi is approximately 1 mm, which is almost twice long as humans [73].

Another prominent feature is the gut-associated lymphoid tissue, the largest immunological organ in the body, representing the largest component of the mucosal immune system. Its role is to sample the luminal antigens via microfold (M) cells, which make specific inductive sites, so-called Peyer patches [78].

### 6.2. Enterocytes

Enterocytes form a monolayer of cells, the intestinal surface, with its luminal side orientated to the external environment. Enterocytes are polarized cells whose membrane on the luminal side forms a microvillar membrane, the so-called “brush-border”, while on the lateral side, enterocytes are in tight connection with each other forming a monolayer. At the basal side, enterocytes are in contact with the submucosa consisting of the heterogeneous populations of the immune cells within a connective tissue matrix. Thus, the intestinal surface represents both an absorptive surface and a barrier and these simultaneous processes are regulated by enterocytes. As a part of the absorptive and digestive machinery, enterocytes possess enzymes on their surface, which perform the final breakage of peptides and polysaccharides, making them ready for absorption. However, not only can amino acids and small peptides reach the enterocytes brush-border, but also lesser amounts of intact proteins that are antigenic. These gastric-digestion-resistant proteins/antigens are endocytosed by enterocytes, which cleave them within the lysosomal compartment and hence prevent their penetration and contact with the lamina propria [79]. To fulfill their important role as a natural barrier, enterocytes form a strong connection with adjacent enterocytes such as apical junctional complexes composed of tight and adherent junctions and subjacent desmosomes. These junctions are especially important, and their proper structure and disposition are required for preventing paracellular leakage of antigens and other molecules through the epithelium. It is interesting that the plant-derived food cysteine protease, actinidase, increased intestinal permeability in mice and an in vitro human Caco-2 cell culture model by proteolytical degradation of the key epithelial tight junction transmembrane protein, occludin [80]. Furthermore, this protease induced release of pro-inflammatory cytokines interleukin (IL)-1β, tumor necrosis factor (TNF) α, and IL-33 in mouse-derived intestinal organoids [81]. Previous findings indicated that food allergens are involved in the sensitization process in food allergy pathogenesis by challenging enterocytes and compromising the epithelial barrier.

Previously mentioned microvilli positioned on the luminal side of enterocytes possess integral mucin-like glycoproteins in the membrane. These glycoproteins form the glycocalyx, a continuous and thick layer that contains intramembrane enzymes involved in the terminal digestion of glycoproteins but the glycocalyx has also been shown to adsorb pancreatic enzymes [82].

In addition, enterocytes represent a hub for innate and adaptive immune reactions [83]. The key players in innate immune responses and sensors of predominantly commensal but also pathogen-associated molecular patterns (PAMPs) are Toll-like receptors (TLRs). As a part of the adaptive immune response, enterocytes possess low-affinity IgE receptors called CD23 markers (discussed in detail in Section 6.2.1). Various TLRs are expressed in mice, designated as TLR2, TLR4, and TLR5, while TLR9 is expressed in mice enterocytes with conventional flora but not in germ-free mice [84]. Moreover, it has been shown that TLR signaling is essential in the maintenance of the functional epithelial barrier, which is reflected in their involvement in the epithelial cell proliferation, maintenance of tight junctions, IgA production, etc. [84].

As already mentioned, enterocytes have been considered as antigen-presenting cells since they constitutively express a low level of MHC class II molecules. This implies that enterocytes can present soluble antigens to antigen-primed T cells. Furthermore, under constant inflammation, the stimuli level of MHC class II molecules increases [85]. Enterocytes are very dynamic cells and in constant contact with adjacent cells and tissues, actively communicating with T-cells, DCs, granulocytes, monocytes, macrophages, and mast cells [86].

#### 6.2.1. Low-Affinity IgE Receptor, CD23, as Molecular Evidence of Enterocyte Involvement in the Modulation of Food Allergy Responses

CD23 or FcεRII is a so-called “low-affinity” IgE receptor, first discovered on human B lymphocytes [87]. The molecule of CD23 exists as a homotrimer, with the IgE binding domain belonging to the C-type lectin superfamily and connected to the membrane by a triple α-helical coiled-coil stem region; it exists as a membrane-bound form and as soluble fragments that are released by cleavage in the stalk region [88].

**Figure 1:**
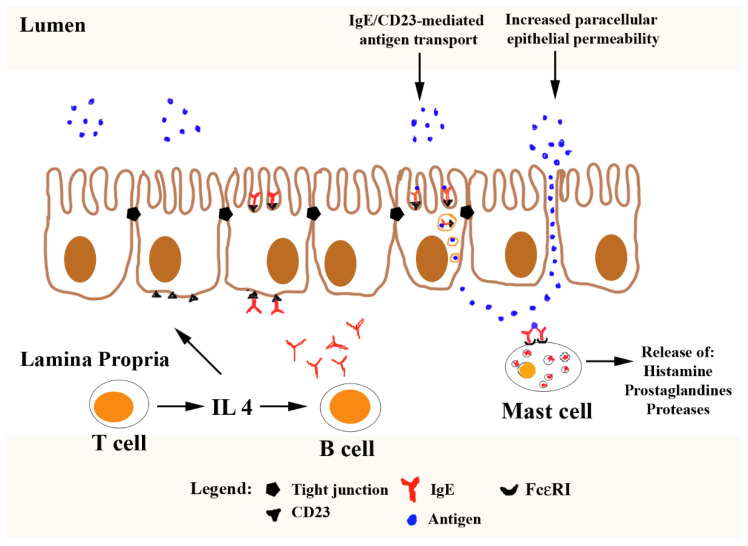
The increased transepithelial transport of allergen mediated by IgE/CD23. IL-4 synthesis by T cells induces isotype switch and production of IgE by B cells. IL-4 is secreted into the serum and intestinal lumen and acts on enterocytes to upregulate the expression of the low-affinity IgE receptor, CD23 (FcεRII). Further, IgE bound to high-affinity receptor FcεRI on the mast cell surface and CD23 and consequently, the large amount of antigen can cross the epithelial barrier and bypass the degradation machinery. Adopted and modified from Yu [91].

The transport of the allergens from the lumen of the intestine to the underlying immune system occurs by several routes: by paracellular diffusion, via M cells in Peyer’s patches, via goblet cell-associated antigen passage, and by enterocyte transcytosis [89]. The uptake of allergens by enterocytes can occur as an active process after stimuli from the pre-existing allergen-specific IgA and IgE antibodies because enterocytes have a low-affinity IgE receptor, CD23 [90]. Increased transport of a specific allergen in mice, which is actively or passively sensitized, was noted upon luminal challenge [38]. This increased transepithelial transport of allergen was mediated by IgE/CD23 via IgE depletion and anti-CD23 antibody reduced epithelial uptake of antigen (Figure 1). Phase I of enhanced transepithelial transport of antigen was mediated by IL-4 regulating IgE synthesis and expression of CD23 on the enterocytes. Therefore, the large amount of antigen that crosses the epithelial barrier and bypasses the degradation machinery, can be a consequence of antigen binding to IgE/CD23 in the enterocytes [38].

#### 6.2.2. Proteomics in Revealing Extraordinary Plasticity of Aging and Differently Stimulated Enterocyte Monolayer

The renewal of the intestinal epithelium is dependent on highly proliferative intestinal stem cells that give rise to multiple differentiated cells of the absorptive and secretory types, such as enterocytes and goblet cells. In a study by Gebert, et al. [92], mass-spectrometry-based proteomics was used to compare proteomes of intestinal crypts between mice of different age groups to reveal age- and region-specific differences and investigate young and old mice responses to dietary changes [92]. Aside from the major clues of this study, lateral findings related to the comparison of proteomes of mice infected with bacteria and geriatric mice populations are relevant for understanding the role of enterocytes in the complex network of oral tolerance and its break.

The impact of aging on intestinal crypts was revealed by comparing the abundances of over 5000 proteins between young, old, and geriatric mice. Approximately 250 proteins were identified as affected by aging in the geriatric mice: increased levels of proteins related to the immune response to pathogens, including antimicrobial peptides, Paneth cell markers, MHC proteins (H2-Aa and H2-Ab1), and lectins as inflammatory cytokines known to increase with aging [92]. In addition, decreased levels of stem cell markers, proteins involved in DNA replication and cell-cycle progression, and many more were found. Due to the increased levels of immune response-related proteins, the authors compared aging proteome profiles to single-cell RNA-sequence data describing the response of the intestinal epithelium to bacterial infection by *Salmonella enterica* and found that genes upregulated in response to bacteria related to the immune response, explain 33% of all the proteins upregulated in the geriatric mice population. Importantly, most of the changes in inflammation-related proteins, as well as an increase in mucosal immunoglobulins, were already noticed in old mice [92].

## 7. Conclusions

The science, which is mostly human-centered, has come a long way since the recognition of the enterocytes as not only the barrier and main absorptive cells, but also as the cells actively involved in immunological responses, utilizing mostly various animal models, from mice to large animals, such as sheep and pigs. However, many of these immune responses and enterocytes’ roles are still to be discovered. Perhaps, the most promising direction or source of novel immunological roles of enterocytes are dedicated and comprehensive transcriptomic and proteomic studies dealing with the ever-changing plasticity of enterocyte proteomes under various stimuli or within different age frames. Substantial knowledge of hypersensitivity food reactions in higher animals was corroborated from numerous animal studies aimed to understand adverse food reactions in the human population, whose results are being extrapolated for people. A significantly lower number of dedicated studies, whose primary aim was to understand animal food hypersensitivity, suggested that mammals and humans do share many features of food allergies, besides differences immanent to mammals solely. Behind the very complex anatomy of the intestine, the only constants are changes and preparedness to react and recognize the stimuli in a very loaded and dynamic environment. It seems that enterocytes, in both mammals or humans, occupy the central position and possess multiple roles in the interplay responsible for oral sensitization, oral tolerance, and its break.

## Figures and Tables

**Table 1:** General classification of animal food models with proposed references for further reading.

Animal Models of Food Allergy					
Small Animal Models			Large Animal Models (Non-Murine Models)		
Murine Models		Zebrafish model	Dog Model	Pig Model	Sheep Model
Mice strains	Rat strains				
BALB/c; C57BL/6; A/J; C3H/HcJ; Liu, Navarro and Lopata [24]; Gonipeta, Kim and Gangur [25]	SD; Wistar; BN; Knippels and Penninks [26]; Behroo [27]	Contreras, et al. [28]; Bailone, et al. [29]; Fuentes-Appelgren, et al. [30]	Ermel, et al. [31]; Buchanan and Frick [32]	Helm, et al. [33]; Rupa, et al. [34]	Gramberg, Veer, Hehir, Meeusen and Bischof [21]

**Table 2:** Overview of the studies based on murine and non-murine models from which the knowledge on the involvement of enterocytes in food allergic reactions was gained.

Murine Models (Mice and Rat Strains)	Allergenic Food Source/ Allergen	Findings of Enterocytes Involvement	References
Wistar male rats	Horseradish peroxidase	Increased antigen uptake was observed within the endosomal compartment of jejunal enterocytes in sensitized rats before mast cell activation	Berin, et al. [37]
BALB/c mice	Horseradish peroxidase	IL-4 regulates IgE/CD23-mediated enhanced transepithelial antigen transport in the sensitized mouse intestine.	Yu, et al. [38]
C3H/He mice	Ovalbumin (digested)	Epithelial exosomes are antigen-presenting vesicles bearing MHC class II/peptide complexes that prime for an immunogenic response rather than tolerogenic in the systemic challenge.	Van Niel, et al. [39]
Sprague-Dawley rats	Ovalbumin and horseradish peroxidase	Translocations of CD23 from the cell surface to the membrane of allergen-containing endosomes, confirms the internalization of CD23 protein upon luminal antigen challenge in the sensitized intestine.	Yang, et al. [40]
BALB/c mice	Ovalbumin used as antigen	Postprandial chylomicron formation profoundly affects the absorption and systemic dissemination of dietary antigens. The fat content of a meal may affect immune responses to dietary antigens by modulating antigen absorption and transport.	Wang, et al. [41]
BALB/c mice	Ovalbumin used as antigen	Vasoactive intestinal peptides deficient mice failed to induce type 1 regulatory T cells in the intestine and retarded the establishment of antigen (Ag)-specific immune tolerance.	Zeng, et al. [42]
C57BL/6J mice	n/a ^#^	Intestinal intraepithelial lymphocyte to enterocyte crosstalk regulates the production of bactericidal angiogenin 4 by Paneth cells upon microbial challenge.	Walker, et al. [43]
C3H/HeOuJ and C57Bl-6 mice (males)	n/a ^#^	A novel link between remote injury and enterocyte TLR4 signaling leading to barrier injury, potentially through HMGB1 as a ligand, demonstrating the reversal of adverse effects through activation of TLR9.	Sodhi, et al. [44]
C3H/HeJ mice	peanut butter protein	Dietary medium-chain triglycerides promote allergic sensitization and anaphylaxis by affecting antigen absorption and availability and by stimulating T_H_2 responses.	Li, et al. [45]
Female Balb/C mice	Horseradish peroxidase	bDelta5 slice form of low-affinity CD23 receptor mediates the apical to basolateral transport of free IgE, whereas classical b from is much more efficient in mediating the transcytosis of IgE/allergen complexes.	Montagnac, et al. [46]
Wiskott-Aldrich syndrome Balb/C mice	Ovalbumin& Horseradish peroxidase	Spontaneous sensitization and intestinal allergy occurred independently of FcεRI expression on mast cells and basophils. results imply that therapeutic targeting of the IgE/FcεRI activation cascade will not affect sensitization to food.	Lexmond, et al. [47]

^#^ Not applicable.

## References

[1] Sicherer S.H., Sampson H.A. (2010). Food allergy. J. Allergy Clin. Immunol..

[2] Cianferoni A., Spergel J.M. (2009). Food allergy: Review, classification and diagnosis. Allergol. Int..

[3] Miller W.H., Griffin C.E. (2012). Muller and Kirk’ s Small Animal Dermatology.

[4] Luckschander N., Allenspach K., Hall J., Seibold F., Gröne A., Doherr G.M., Gaschen F. (2006). Perinuclear antineutrophilic cytoplasmic antibody and response to treatment in diarrheic dogs with food responsive disease or inflammatory bowel disease. J. Vet. Intern. Med..

[5] Crowe S.E., Perdue M.H. (1992). Gastrointestinal food hypersensitivity: Basic mechanisms of pathophysiology. Gastroenterology.

[6] Favrot C., Steffan J., Seewald W., Picco F. (2010). A prospective study on the clinical features of chronic canine atopic dermatitis and its diagnosis. Vet. Dermatol..

[7] Jackson H.A., Jackson M.W., Coblentz L., Hammerberg B. (2003). Evaluation of the clinical and allergen specific serum immunoglobulin E responses to oral challenge with cornstarch, corn, soy and a soy hydrolysate diet in dogs with spontaneous food allergy. Vet. Dermatol..

[8] Ognjenovic J., Milcic-Matic N., Smiljanic K., Vuckovic O., Burazer L., Popovic N., Stanic-Vucinic D., Velickovic T.C. (2013). Immunoproteomic characterization of Ambrosia artemisiifolia pollen allergens in canine atopic dermatitis. Vet. Immunol. Immunopathol..

[9] Pedersen N.C. (1999). A review of immunologic diseases of the dog. Vet. Immunol. Immunopathol..

[10] Olivry T., Mueller R.S. (2019). Critically appraised topic on adverse food reactions of companion animals (7): Signalment and cutaneous manifestations of dogs and cats with adverse food reactions. BMC Vet. Res..

[11] Mueller R.S., Olivry T. (2018). Critically appraised topic on adverse food reactions of companion animals (6): Prevalence of noncutaneous manifestations of adverse food reactions in dogs and cats. BMC Vet. Res..

[12] Olivry T., Mueller R.S. (2017). Critically appraised topic on adverse food reactions of companion animals (3): Prevalence of cutaneous adverse food reactions in dogs and cats. BMC Vet. Res..

[13] Mueller R.S., Olivry T. (2017). Critically appraised topic on adverse food reactions of companion animals (4): Can we diagnose adverse food reactions in dogs and cats with in vivo or in vitro tests?. BMC Vet. Res..

[14] Guilford W.G., Jones B.R., Markwell P.J., Arthur D.G., Collett M.G., Harte J.G. (2001). Food sensitivity in cats with chronic idiopathic gastrointestinal problems. J. Vet. Intern. Med..

[15] Olivry T., Mueller R.S., Prélaud P. (2015). Critically appraised topic on adverse food reactions of companion animals (1): Duration of elimination diets. BMC Vet. Res..

[16] Olivry T., Mueller R.S. (2020). Critically appraised topic on adverse food reactions of companion animals (9): Time to flare of cutaneous signs after a dietary challenge in dogs and cats with food allergies. BMC Vet. Res..

[17] Pali-Schöll I., De Lucia M., Jackson H., Janda J., Mueller R.S., Jensen-Jarolim E. (2017). Comparing immediate-type food allergy in humans and companion animals-revealing unmet needs. Allergy.

[18] Aldemir H., Bars R., Herouet-Guicheney C. (2009). Murine models for evaluating the allergenicity of novel proteins and foods. Regul. Toxicol. Pharmacol..

[19] Knippels L.M.J., Penninks A.H., Meeteren M.V. (1999). Humoral and Cellular Immune Responses in Different Rat Strains on Oral Exposure to Ovalbumin. Food Chem. Toxicol..

[20] Radcliffe J.S., Brito L.F., Reddivari L., Schmidt M., Herman E.M., Schinckel A.P. (2019). A swine model of soy protein–induced food allergenicity: Implications in human and swine nutrition. Anim. Front..

[21] Gramberg J.L.V., Veer M.J.D., Hehir R.E.O., Meeusen E.N.T., Bischof R.J. (2012). Induction of Allergic Responses to Peanut Allergen in Sheep. PLoS ONE.

[22] Teuber S.S., Del Val G., Morigasaki S., Jung H.R., Eisele P.H., Frick O.L., Buchanan B.B. (2002). The atopic dog as a model of peanut and tree nut food allergy. J. Allergy Clin. Immunol..

[23] Santoro D., Marsella R. (2014). Animal Models of Allergic Diseases. Vet. Sci..

[24] Liu T., Navarro S., Lopata A.L. (2016). Current advances of murine models for food allergy. Mol. Immunol..

[25] Gonipeta B., Kim E., Gangur V. (2015). Mouse models of food allergy: How well do they simulate the human disorder?. Crit. Rev. Food Sci. Nutr..

[26] Knippels L.M., Penninks A.H. (2005). Recent advances using rodent models for predicting human allergenicity. Toxicol. Appl. Pharmacol..

[27] Behroo L. (2015). Fascinating Findings from Sensitizing the Wistar Strain Rats Recruited as Peanut-Allergy Model. EC Nutr..

[28] Contreras M., Pacheco I., Alberdi P., Díaz-Sánchez S., Artigas-Jerónimo S., Mateos-Hernández L., Villar M., Cabezas-Cruz A., de la Fuente J. (2020). Allergic Reactions and Immunity in Response to Tick Salivary Biogenic Substances and Red Meat Consumption in the Zebrafish Model. Front. Cell Infect. Microbiol..

[29] Bailone R.L., de Aguiar L.K., de Oliveira Roca R., Borra R.C., Corrêa T., Janke H., Fukushima H.C.S. (2019). Zebrafish as an animal model for food safety research: Trends in the animal research. Food Biotechnol..

[30] Fuentes-Appelgren P., Opazo R., Barros L., Feijoó C.G., Urzúa V., Romero J. (2014). Effect of the dietary inclusion of soybean components on the innate immune system in zebrafish. Zebrafish.

[31] Ermel R.W., Kock M., Griffey S.M., Reinhart G.A., Frick O.L. (1997). The atopic dog: A model for food allergy. Lab. Anim. Sci..

[32] Buchanan B., Frick O. (2002). The Dog as a Model for Food Allergy. Ann. N. Y. Acad. Sci..

[33] Helm R.M., Furuta G.T., Stanley J.S., Ye J., Cockrell G., Connaughton C., Simpson P., Bannon G.A., Burks A.W. (2002). A neonatal swine model for peanut allergy. J. Allergy Clin. Immunol..

[34] Rupa P., Hamilton K., Cirinna M., Wilkie B.N. (2008). A neonatal swine model of allergy induced by the major food allergen chicken ovomucoid (Gal d 1). Int. Arch. Allergy Immunol..

[35] Kanagaratham C., Sallis B.F., Fiebiger E. (2018). Experimental Models for Studying Food Allergy. Cell. Mol. Gastroenterol. Hepatol..

[36] Huang J., Liu C., Wang Y., Wang C., Xie M., Qian Y., Fu L. (2018). Application of in vitro and in vivo models in the study of food allergy. Food Sci. Hum. Wellness.

[37] Berin M.C., Kiliaan A.J., Yang P.C., Groot J.A., Taminiau J.A., Perdue M.H. (1997). Rapid transepithelial antigen transport in rat jejunum: Impact of sensitization and the hypersensitivity reaction. Gastroenterology.

[38] Yu L.C.H., Yang P.C., Berin M.C., Di Leo V., Conrad D.H., McKay D.M., Satoskar A.R., Perdue M.H. (2001). Enhanced transepithelial antigen transport in intestine of allergic mice is mediated by IgE/CD23 and regulated by interleukin-4. Gastroenterology.

[39] Van Niel G., Mallegol J., Bevilacqua C., Candalh C., Brugière S., Tomaskovic-Crook E., Heath J.K., Cerf-Bensussan N., Heyman M. (2003). Intestinal epithelial exosomes carry MHC class II/peptides able to inform the immune system in mice. Gut.

[40] Yang P.C., Berin M.C., Yu L.C., Conrad D.H., Perdue M.H. (2000). Enhanced intestinal transepithelial antigen transport in allergic rats is mediated by IgE and CD23 (FcepsilonRII). J. Clin. Investig..

[41] Wang Y., Ghoshal S., Ward M., de Villiers W., Woodward J., Eckhardt E. (2009). Chylomicrons Promote Intestinal Absorption and Systemic Dissemination of Dietary Antigen (Ovalbumin) in Mice. PLoS ONE.

[42] Zeng H.T., Liu J.Q., Zhao M., Yu D., Yang G., Mo L.H., Liu Z.Q., Wang S., Liu Z.G., Yang P.C. (2020). Exosomes carry IL-10 and antigen/MHC II complexes to induce antigen-specific oral tolerance. Cytokine.

[43] Walker C.R., Hautefort I., Dalton J.E., Overweg K., Egan C.E., Bongaerts R.J., Newton D.J., Cruickshank S.M., Andrew E.M., Carding S.R. (2013). Intestinal Intraepithelial Lymphocyte-Enterocyte Crosstalk Regulates Production of Bactericidal Angiogenin 4 by Paneth Cells upon Microbial Challenge. PLoS ONE.

[44] Sodhi C., Levy R., Gill R., Neal M.D., Richardson W., Branca M., Russo A., Prindle T., Billiar T.R., Hackam D.J. (2011). DNA attenuates enterocyte Toll-like receptor 4-mediated intestinal mucosal injury after remote trauma. Am. J. Physiol. -Gastrointest. Liver Physiol..

[45] Li J., Wang Y., Tang L., de Villiers W.J.S., Cohen D., Woodward J., Finkelman F.D., Eckhardt E.R.M. (2013). Dietary medium-chain triglycerides promote oral allergic sensitization and orally induced anaphylaxis to peanut protein in mice. J. Allergy Clin. Immunol..

[46] Montagnac G., Yu L.C., Bevilacqua C., Heyman M., Conrad D.H., Perdue M.H., Benmerah A. (2005). Differential role for CD23 splice forms in apical to basolateral transcytosis of IgE/allergen complexes. Traffic.

[47] Lexmond W.S., Goettel J.A., Sallis B.F., McCann K., Rings E.H.H.M., Jensen-Jarolim E., Nurko S., Snapper S.B., Fiebiger E. (2017). Spontaneous food allergy in Was−/− mice occurs independent of FcεRI-mediated mast cell activation. Allergy.

[48] Faria A.M.C., Weiner H.L. (2005). Oral tolerance. Immunol. Rev..

[49] Tordesillas L., Gómez-Casado C., Garrido-Arandia M., Murua-García A., Palacín A., Varela J., Konieczna P., Cuesta-Herranz J., Akdis C.A., O’Mahony L. (2013). Transport of Pru p 3 across gastrointestinal epithelium—An essential step towards the induction of food allergy?. Clin. Exp. Allergy.

[50] Knoop K.A., Miller M.J., Newberry R.D. (2013). Transepithelial antigen delivery in the small intestine: Different paths, different outcomes. Curr. Opin. Gastroenterol..

[51] Verhasselt V. (2010). Oral tolerance in neonates: From basics to potential prevention of allergic disease. Mucosal Immunol..

[52] Thorstenson K.M., Khoruts A. (2001). Generation of anergic and potentially immunoregulatory CD25+CD4 T cells in vivo after induction of peripheral tolerance with intravenous or oral antigen. J. Immunol..

[53] Tordesillas L., Berin M.C. (2018). Mechanisms of Oral Tolerance. Clin. Rev. Allergy Immunol..

[54] McDole J.R., Wheeler L.W., McDonald K.G., Wang B., Konjufca V., Knoop K.A., Newberry R.D., Miller M.J. (2012). Goblet cells deliver luminal antigen to CD103+ dendritic cells in the small intestine. Nature.

[55] Awasthi A., Carrier Y., Peron J.P., Bettelli E., Kamanaka M., Flavell R.A., Kuchroo V.K., Oukka M., Weiner H.L. (2007). A dominant function for interleukin 27 in generating interleukin 10-producing anti-inflammatory T cells. Nat. Immunol..

[56] Chen Y., Inobe J., Kuchroo V.K., Baron J.L., Janeway C.A., Weiner H.L. (1996). Oral tolerance in myelin basic protein T-cell receptor transgenic mice: Suppression of autoimmune encephalomyelitis and dose-dependent induction of regulatory cells. Proc. Natl. Acad. Sci. USA.

[57] Cahenzli J., Köller Y., Wyss M., Geuking M.B., McCoy K.D. (2013). Intestinal microbial diversity during early-life colonization shapes long-term IgE levels. Cell Host Microbe.

[58] Atarashi K., Tanoue T., Oshima K., Suda W., Nagano Y., Nishikawa H., Fukuda S., Saito T., Narushima S., Hase K. (2013). Treg induction by a rationally selected mixture of Clostridia strains from the human microbiota. Nature.

[59] Zimmer K.P., Buning J., Weber P., Kaiserlian D., Strobel S. (2000). Modulation of Antigen Trafficking to MHC Class II–Positive Late Endosomes of Enterocytes. Gatroenterology.

[60] Kucuk Z.Y., Strait R., Khodoun M.V., Mahler A., Hogan S., Finkelman F.D. (2012). Induction and suppression of allergic diarrhea and systemic anaphylaxis in a murine model of food allergy. J. Allergy Clin. Immunol..

[61] Lönnroth I., Holmgren J. (1973). Subunit structure of cholera toxin. J. Gen. Microbiol.

[62] Snider D.P., Marshall J.S., Perdue M.H., Liang H. (1994). Production of IgE antibody and allergic sensitization of intestinal and peripheral tissues after oral immunization with protein Ag and cholera toxin. J. Immunol..

[63] Yamamoto M., Kiyono H., Kweon M.N., Yamamoto S., Fujihashi K., Kurazono H., Imaoka K., Bluethmann H., Takahashi I., Takeda Y. (2000). Enterotoxin adjuvants have direct effects on T cells and antigen-presenting cells that result in either interleukin-4-dependent or-independent immune responses. J. Infect. Dis..

[64] Brunner R., Wallmann J., Szalai K., Karagiannis P., Kopp T., Scheiner O., Jensen-Jarolim E., Pali-Schöll I. (2007). The impact of aluminium in acid-suppressing drugs on the immune response of BALB/c mice. Clin. Exp. Allergy.

[65] Yang P.C., Jury J., Söderholm J.D., Sherman P.M., McKay D.M., Perdue M.H. (2006). Chronic psychological stress in rats induces intestinal sensitization to luminal antigens. Am. J. Pathol..

[66] Kean D.E., Goodridge H.S., McGuinness S., Harnett M.M., Alcocer M.J.C., Harnett W. (2006). Differential Polarization of Immune Responses by Plant 2S Seed Albumins, Ber e 1, and SFA8. J. Immunol..

[67] Martos G., Lopez-Exposito I., Bencharitiwong R., Berin M.C., Nowak-Węgrzyn A. (2011). Mechanisms underlying differential food allergy response to heated egg. J. Allergy Clin. Immunol..

[68] Radosavljevic J., Nordlund E., Mihajlovic L., Krstic M., Bohn T., Buchert J., Velickovic T.C., Smit J. (2014). Sensitizing potential of enzymatically cross-linked peanut proteins in a mouse model of peanut allergy. Mol. Nutr. Food Res..

[69] Mihajlovic L., Radosavljevic J., Nordlund E., Krstic M., Bohn T., Smit J., Buchert J., Cirkovic Velickovic T. (2016). Peanut protein structure, polyphenol content and immune response to peanut proteins in vivo are modulated by laccase. Food Funct..

[70] Dwinell M.B., Johanesen P.A., Smith J.M. (2003). Immunobiology of epithelial chemokines in the intestinal mucosa. Surgery.

[71] Steele L., Mayer L., Berin M.C. (2012). Mucosal immunology of tolerance and allergy in the gastrointestinal tract. Immunol. Res..

[72] Vitale S., Picascia S., Gianfrani C. (2016). The cross-talk between enterocytes and intraepithelial lymphocytes. Mol. Cell Pediatr..

[73] Hall E.J., German A.J., Willard M.D., Lappin M.R., Cave N., Washabau R.J., Bergman P.J. (2012). Small Intestine. Canine and Feline Gastroenterology.

[74] Shanahan F. (2002). The host-microbe interface within the gut. Bailliere’s Best Pract. Res. Clin. Gastroenterol..

[75] Macia L., Tan J., Vieira A.T., Leach K., Stanley D., Luong S., Maruya M., Ian McKenzie C., Hijikata A., Wong C. (2015). Metabolite-sensing receptors GPR43 and GPR109A facilitate dietary fibre-induced gut homeostasis through regulation of the inflammasome. Nat. Commun.

[76] Kittana H., Gomes-Neto J.C., Heck K., Geis A.L., Segura Muñoz R.R., Cody L.A., Schmaltz R.J., Bindels L.B., Sinha R., Hostetter J.M. (2018). Commensal Escherichia coli Strains Can Promote Intestinal Inflammation via Differential Interleukin-6 Production. Front. Immunol..

[77] Corfield A.P., Carroll D., Myerscough N., Probert C.S.J. (2001). Mucins in the gastrointestinal tract in health and disease. Front. Biosci..

[78] Butler J.E., Sinkora M. (2013). The enigma of the lower gut-associated lymphoid tissue (GALT). J. Leukoc. Biol..

[79] Fujita M., Reinhart F., Neutrat M. (1988). Convergence of apical and basolateral endocytic pathways at apical late endosomes in absorptive cells of suckling rat ileum in vivo. J. Cell Sci..

[80] Grozdanovic M.M., Čavić M., Nešić A., Andjelković U., Akbari P., Smit J.J., Gavrović-Jankulović M. (2016). Kiwifruit cysteine protease actinidin compromises the intestinal barrier by disrupting tight junctions. Biochim. Biophys. Acta-Gen. Subj..

[81] Nešić A., Stam A., Čavić M., Ten Klooster J.P., Pieters R., Smit J., Gavrović-Jankulović M. (2019). Activation of epithelial cells by the major kiwifruit allergen Act d 1 in human and mouse-derived intestinal model. J. Funct. Foods.

[82] Mestecky J. (2005). Mucosal Immunology.

[83] Miron N., Cristea V. (2012). Enterocytes: Active cells in tolerance to food and microbial antigens in the gut. Clin. Exp. Immunol..

[84] Abreu M.T. (2010). Toll-like receptor signalling in the intestinal epithelium: How bacterial recognition shapes intestinal function. Nat. Rev. Immunol..

[85] Steiniger B., Falk P., Lohmuller M., Van Der Meide P.H. (1989). Class II MHC antigens in the rat digestive system. Normal distribution and induced expression after interferon-gamma treatment in vivo. Immunology.

[86] Shaykhiev R., Bals R. (2007). Interactions between epithelial cells and leukocytes in immunity and tissue homeostasis. J. Leukoc. Biol..

[87] Conrad D.H. (1990). Fc epsilon RII/CD23: The low affinity receptor for IgE. Annu. Rev. Immunol..

[88] Sutton B.J., Davies A.M., Sutton B.J. (2015). Structure and dynamics of IgE—receptor interactions: Fc e RI and CD23 / Fc e RII. Immunol. Rev..

[89] Mallegol J., Van Niel G., Lebreton C., Lepelletier Y., Candalh C., Dugave C., Heath J.K., Raposo G., Cerf-Bensussan N., Heyman M. (2007). T84-intestinal epithelial exosomes bear MHC class II/peptide complexes potentiating antigen presentation by dendritic cells. Gastroenterology.

[90] Tu Y., Salim S.A., Bourgeois J., Di Leo V., Irvine E.J., Marshall J.K., Perdue M.H. (2005). CD23-mediated IgE transport across human intestinal epithelium: Inhibition by blocking sites of translation or binding. Gastroenterology.

[91] Yu L.C.-H. (2012). Intestinal Epithelial Barrier Dysfunction in Food Hypersensitivity. J. Allergy.

[92] Gebert N., Cheng C.-W., Kirkpatrick J.M., Di Fraia D., Yun J., Schädel P., Pace S., Garside G.B., Werz O., Rudolph K.L. (2020). Region-Specific Proteome Changes of the Intestinal Epithelium during Aging and Dietary Restriction. Cell Rep..

